# The Decrease of n-3 Fatty Acid Energy Percentage in an Equicaloric Diet Fed to B6C3Fe Mice for Three Generations Elicits Obesity

**DOI:** 10.1155/2009/867041

**Published:** 2009-09-16

**Authors:** Ingeborg Hanbauer, Ignacio Rivero-Covelo, Ekrem Maloku, Adam Baca, Qiaoyan Hu, Joseph R. Hibbeln, John M. Davis

**Affiliations:** ^1^Department of Psychiatry, School of Medicine University of Illinois at Chicago, 1601 W. Taylor Street, Chicago, IL 60612, USA; ^2^National Institutes on Alcohol Abuse and Alcoholism, NIH, Bethesda, MD 20892-9410, USA

## Abstract

Feeding mice, over 3 generations, an equicaloric diet in which *α*-linolenic acid, the dietary precursor of n-3 polyunsaturated fatty acids, was substituted by linoleic acid, the dietary precursor of n-6 polyunsaturated fatty acids, significantly increased body weight throughout life when compared with standard diet-fed mice. Adipogenesis observed in the low *n*-3 fatty acid mice was accompanied by a 6-fold upregulation of stearyl-coenzyme A desaturase 1 (Scd1), whose activity is correlated to plasma triglyceride levels. In total liver lipid and phospholipid extracts, the sum of n-3 fatty acids and the individual longer carbon chain acids, eicosapentaenoic acid (20:5n3), docosapentaenoic acid (22:5n3), and docosahexaenoic acid (22:6n3) were significantly decreased whereas arachidonic acid (20:4n6) was significantly increased. In addition, low *n*-3 fatty acid-fed mice had liver steatosis, heart, and kidney hypertrophy. Hence, reducing dietary *α*-linolenic acid, from 1.02 energy % to 0.16 energy % combined with raising linoleic acid intake resulted in obesity and had detrimental consequences on organ function.

## 1. Introduction

Obesity is generally associated with a lifestyle where caloric energy intake exceeds energy expenditure. Increased fat and carbohydrate consumption creates a scenario for increased energy intake and storage of superfluous energy in adipose tissue. Pre-existing adipocytes can accumulate fatty acids as triglycerides resulting in adipose tissue expansion. On the other hand, adipose tissue mass may also be increased by *de novo* adipocyte differentiation [1–3]. In fact, it has been shown that nutritional or environmental changes can alter the equilibrium between adipocyte and preadipocyte populations [4]. In this context, Ailhaud et al. [1] proposed in a rodent model that excessive consumption of dietary n-6 fatty acid (FA)s with insufficient amounts of n-3 FAs might become another risk factor for obesity. To this date, little is known on the pathophysiological consequences of a disproportional intake of polyunsaturated fatty acids. 

To obtain a model for “Western” diets that may still be used in many human cohorts we chose to feed mice, for at least three generation, an equicaloric diet which had disproportionate n-6 FA/n-3 FA ratio and a cholesterol level that was higher than in a vegetarian diet but below atherogenic level. Here, we report a serendipitous finding of obesity in offspring of mice fed a safflower oil-based diet (low n-3 diet, Harlan Teklad, TK 00522) over three generations in comparison to an equicaloric soy and soy oil-based diet (standard n-3, Harlan Teklad, TD 7912). Neither diet contained long chain omega-3 nor omega-6 fats detectable in direct compositional analysis, thus the 18 carbon precursors were the sources of essential fatty acids (Table 1). The low n-3 diet had lower *α*-linolenic acid (0.16 energy % versus 1.02 energy %) and higher linoleic acid (12.31 energy % versus 9.68 energy %) compared to the standard n-3 diet. As shown in Table 1, the low n-3 diet had similar energy derived from protein and fats and had only slightly higher energy (3.4 %) from carbohydrates compared to the standard n-3 diet. The energy percentage contributed by saturated and monounsaturated fatty acids was 21% and 28% lower in the low n-3, compared to the standard n-3 diet.

## 2. Obesity in Mice Fed a Low-Energy % n-3 FA Diet

**Figure 1:**
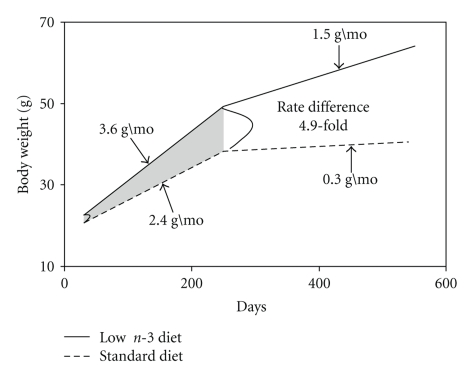
The increase of body weight over time of mice fed the low n-3 fatty acid- or standard rodent diet. A mixed regression model was calculated as described in statistical analysis section. The full line represents the rate of increase in low n-3 fatty acid mice (*n* = 96), and the dashed line represents the rate of increase in standard n-3 fatty acid diet-fed mice (*n* = 76).

The body weight of the third generation of male mice on low n-3 diet was significantly higher than that of their corresponding mates on standard n-3 diet throughout their life. The low n-3 diet group consumed 352.1 ± 10.7 Kcal within a 3-week period whereas mice on standard diet, for the same time period, consumed 336.6 ± 7.1 Kcal. The body weight of both diet groups was measured biweekly, and the rate of increase in body weight was computed using a mixed regression model [5]. Figure 1 shows that low n-3 mice (*n* = 96) gained 3.6 g/mo whereas control mice (*n* = 76) gained 2.4 g/mo from time of weaning to the end of adulthood. In senescent low n-3 mice, the rate of increase in body weight was 1.5 g/month compared to 0.3 g/mo in senescent standard n-3 mice. As pointed out in Figure 1, a 4.9-fold rate difference (*P* = 7.10^−10^) between low n-3 and standard n-3 mice occurred in the senescent group (older than 240 days; open area) in contrast to a 0.5-fold rate difference in the adult group (shaded area). 

**Figure 2:**
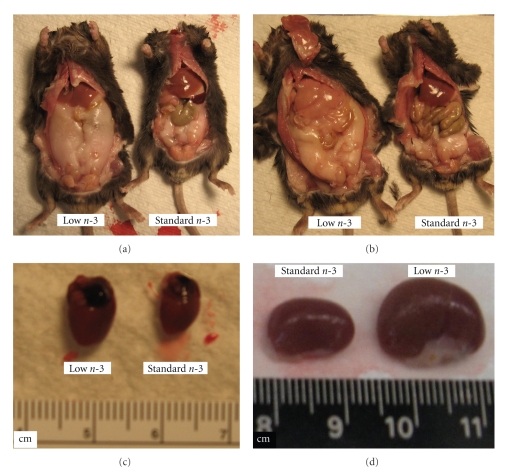
(a) Comparison between a low n-3 fatty acid and a standard n-3 fatty acid diet-fed mouse shows increased inguinal abdominal fat and body length. (b) Comparison of low n-3 fatty acid and a standard diet mouse shows increased body length increased inguinal abdominal fat and liver steatosis (arrow) in the low n-3 fatty acid mouse. (c) Comparison of hearts and (d) comparison of kidneys from low n-3 fatty acid- and standard n-3 fatty acid-fed mice.

 As evident from the data reported in Figure 2, mice on low n-3 diet were prominently larger, and their body length (measured from neck to tail) was strikingly longer than in mice on standard n-3 diet (low n-3 diet: 8.26 ± 0.11 cm (*n* = 10) versus standard n-3 diet: 7.8 ± 0.14 cm, (*n* = 6) *P* < .025). Another striking difference between low n-3 FA-fed and standard n-3 mice was the increased mass of inguinal-abdominal fat (Figures 2(a) and 2(b)) which suggests that lipid metabolism was altered in low n-3 diet-fed mice. A significant increase in weight gain in the third generation of low n-3 mice was noted at 50 days of age, and the onset of inguinal obesity was observed in mice 5 months and older. 

**Figure 3:**
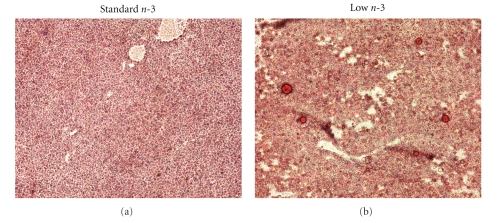
A representative liver section from standard n-3 fatty acid-fed mouse (a), low n-3 fatty acid- (b) and stained with *Oil-red-O* documented liver steatosis.

Adipose tissue is in feedback communication with the liver via adipokines, lipid factors, or lipoproteins. The adipo-hepatic axis, being the link between adipose tissue and liver, is important in regulating the utilization and flux of lipids in both tissues. A deregulation of either one could have detrimental effects on both tissues. In fact, as depicted in Figure 2(b) (arrow), low n-3 FA-fed mice had liver steatosis that was corroborated in *Oil-red-O- *stained liver sections. In the liver of low n-3 mice, a high density of macrovesicular fat droplets is notable while in a control liver slice fat droplets were absent (Figure 3). 

 As pointed out by Tilg and Moschen [6], adipose tissue is not just an energy storage site but it also releases adipocyte-derived mediators that create a link between obesity and inflammatory diseases. As soon as adipocytes get loaded with triglycerides other cells such as endothelial cells, leukocytes, and macrophages invade the adipose tissue. An enhanced crosstalk between macrophages and adipocytes is thought to trigger the release of proinflammatory cytokines and chemokines that might activate a vicious cycle of proinflammatory processes: In this context, we found that in obese low n-3 FA-fed mice hearts (Figure 2(c)) and kidneys (Figure 2(d)) were hypertrophied. A comparison of a representative number of mice per diet group depicted in Table 2 shows that, indeed, organ weights of low n-3 mice were significantly increased. The heart hypertrophy observed in the obese low n-3 mice suggests that these mice might have suffered from complications accompanied by a continuous increase of cardiac work load.

## 3. Changes of Fatty Acid Profile in Liver Total Lipids and Phospholipids

In total lipid and total phospholipid extracts of livers from low n-3 FA-fed mice, the sum of n-3 FA concentrations is significantly lower than in control mice (Table 3). This reduction was also reflected by a decrease of individual fatty acids such as *α*-linolenic acid (18:3n-3) and the longer carbon chain FAs, eicosapentaenoic acid (20:5n-3), docosapentaenoic acid (22:5n-3), and docosahexaenoic acid (22:6n-3). In phospholipid extracts, neither the individual nor the sums of saturated, monounsaturated, linoleic acid (18:2n-6) and higher carbon chain n-6 FA levels were altered by the low n-3 FA diet (Table 3). Instead in total lipid extracts, linoleic acid (18:2n-6) was reduced to 4% of control level whereas arachidonic acid (20:4n-6) was significantly increased. A decrease of linoleic acid concentration in the total liver lipid extract elicited by low n-3 FA diet was also reported by other researchers [7].

Long lasting deprivation of n-3 FA marginally increased the sum of monounsaturated FAs in total liver lipids (Table 3). Since we observed also a tentative increase of individual monounsaturated FAs (data not shown), we studied Scd1, a gene encoding liver stearoyl-coenzyme A desaturase1. Elevation of hepatic stearoyl-coenzyme A desaturase1 activity is correlated to increased plasma triglyceride levels [8]. Dietary and hormonal factors were shown to upregulate liver desaturase activity whereas polyunsaturated FAs were shown to repress desaturase gene upregulation [9]. As shown in Table 4, long lasting low n-3 FA diet elicited a 6.5-fold upregulation of liver stearyl-coenzyme A desaturase 1 (Scd1) gene while Scd2 remained unchanged. Increased SCD1 activity was shown to be rate-limiting in the formation of triglyceride and cholesterol esters [10]. More studies are needed to understand whether liver Scd1 gene was upregulated because n-3 FA levels were too low to repress it or other diet-related changes participated in a more complex gene upregulation. Since monounsaturated FAs are rate-limiting substrates for lipid synthesis in the liver [10], it can be inferred that, the tentative increase of monounsaturated FAs in total lipid extracts shown in Table 3 might be linked to lipogenesis.

## 4. Discussion

In the last decade, research on obesity revealed a redundancy of complex networks involved in the regulation of energy homeostasis. This multitude of regulatory pathways appears to serve the purpose to maintain energy demand during fasting and to store excessive energy during extensive food consumption. The present paper shows that long-lasting n-3 FA deficiency accompanied with n-6 FA excess affects liver function in a manner that may consecutively lead to obesity. One indication for altered energy efficiency set points elicited by long-lasting n-3 FA deficiency is the increase of liver triglyceride synthesis as indicated by fatty liver shown in Figure 2, macrosteatosis shown in Figure 3, and the upregulation of (Scd1), a member of the stearyl-CoA desaturase family shown in Table 4.

Reports in literature showed that hepatic lipogenesis is increased during high carbohydrate or high fat diet [11, 12] but failed to occur in mice with a targeted deletion of SCD1. Since increased SCD1 expression results in increased monounsaturated FA synthesis the tentative increase of the sum of monounsaturated FAs shown in Table 3 may be another indication of increased hepatic lipogenesis. Recently, it was shown that the degree of SCD1 activation correlates with increased plasma triglyceride levels in humans and mice [10]. 

The novel finding of the present paper documents that SCD1 is upregulated by an increased n-6/n-3 FA ratio diet, in contrast to high fat or low fat combined with high carbohydrate diet. Under this circumstance, the downstream events of lipogenesis could be upregulated by decreased n-3 polyunsaturated FA and increased n-6 polyunsaturated FA levels. Hence, in low n-3 mice, the deficit of n-3 FAs, particularly the high carbon chain group (Table 3), might be responsible for the lack of suppression of lipogenic genes. This is in line with findings showing that n-3 poly-unsaturated FAs play an important role in the regulation of lipogenic genes in abdominal fat and brain [13]. Extrapolation of the present data to human populations showed a stark parallel to the increased dietary intake of n-6 FAs in most developed countries in the last 100 years. Due to the competitive relationship between n-3 and n-6 FAs the inevitable increase of the n-6 HUFA pool may irreversibly lead to both obesity and the inflammation resulting in increased mortality [14, 15]. Several epidemiological studies in human cohorts showed that a diet rich in n-6 FA and poor in n-3 FA altered the FA profile in plasma lipids [16, 17]. The outcome of these studies focusing on human cohorts showed that a high-ratio n-6/n-3 FA diet over many generations may have triggered gradual and progressive changes in brain function that could heighten despair or suboptimal social interaction and thus increase the risk of personality disorders [18]. In fact, an increase in severity of acute mania [19] or depressive disorders in elderly [17] were shown to be inversely related to low plasma levels of n-3 polyunsaturated fatty acids. Ongoing research in this laboratory is directed to study the long-term effect of different n-6/n-3 FA ratios in regard to pathophysiological and/or behavioral changes in mice.

## 5. Materials and Methods

### 5.1. Animals

Animals came from our heterozygous mice breeding colony B6C3Fe a/a-Rln^rl^/J (originally obtained from Jackson Laboratories, Bar Harbor, ME, USA). Backcross B6C3Fe male mice were bred in house by pairing male wild type B6C3Fe mice with wild type B6C3Fe dams. The founder strain is not obesity-prone and has no known vulnerabilities to dietary changes in polyunsaturated fatty acids (The Jackson Laboratory Mice Database). Two to five male offspring were housed per cage and had access to their respective diet and water *ad libitum*. One group of the breeder pairs received standard rodent diet (Harlan Teklad 7012, 3.1 Kcal/g) and is also referred to as standard n-3 diet. The second group was fed low n-3 diet throughout three consecutive generations (Harlan Teklad TD.00522, 3.3 Kcal/g, with an addition of 750 ppm cholesterol, as specified by the supplier). This cholesterol concentration is above the range present in “vegetarian” diets (0 − 280 ppm) but is below the range that is considered atherogenic (2000 ppm) for susceptible mouse models [20].

 Pellets from each diet were extracted by a modification of the Folch method [21] using 23 : 0 PC as internal standard. Chloroform extracts were dried under N_2_ and reconstituted in 500 *μ*L of 100 : 3 : 0.3 hexane : MTB : acetic acid. FAs were transmethylated with 14% BF_3_-MeOH at 100°C for 60 minutes and methyl esters were analyzed by gas chromatography as described by Morrison and Smith [22]. 

 The body weight of both diet groups was measured biweekly. In the present study, male wild type mice were used between the ages of two to eight months for biochemical tests. All animal procedures were approved by the University of Illinois at Chicago Animal Care Committee.

### 5.2. Histological Analysis

Livers were quickly excised from carbon dioxide-euthanized mice and frozen on dry ice. Frozen livers were used to prepare sections (5 *μ*m) that were fixed in 10% neutral buffered formalin (10 minutes). After several washes the sections were stained with Oil-Red O for 10 minutes, washed several times with water, and counterstained with hematoxylin for 2 minutes. The sections were mounted with coverslips, observed under a microscope, and photographed.

### 5.3. Liver Fatty Acid Analysis by Gas Chromatography

Seven-month-old mice were decapitated; their livers were rapidly removed and frozen on dry ice. The frozen tissues were subjected to total lipid extraction by a modification of the Folch method using 23 : 0 PC as internal standard. Chloroform extract was dried down under N_2_ and reconstituted in hexane:MTB:acetic acid (100 : 3 : 0.3) for solid phase extraction to separate lipid classes. The total lipid and phospholipids extracts were transmethylated with 14% BF3-methanol and the methyl esters analyzed by gas chromatography as previously described [23].

### 5.4. Liver RNA Isolation and Real-Time PCR

Total RNA was extracted with Trizol (Invitrogen) according to the protocol of the manufacturer. The obtained total RNA pellets were dissolved in RNAse-free water, and possible contaminating DNA was removed using the DNA-free kit (Ambion). The A260/A280 was measured spectrophotometrically. 

 The regulation of Scd1 and Scd2 gene expression was measured by using SYBR green-based real time PCR in liver RNA samples. RNA extraction and quantitative real time PCR (qRT-PCR) were performed at the SABiosciences Service Core, Frederic, MD, based on SABiosciences' ArrayGrade Total RNA Isolation Kit (GA-013), RT2 qPCR primer assays (PPM05664E (scd1), PPM32741A (scd2), and PPM02945A (beta actin)) and RT2 qPCR master mix (PA-011). The mRNA expression level for each gene was normalized against the expression level of the house-keeping gene beta actin within each sample by the comparative Ct (ΔCt) method. The fold-change values were calculated using the ΔΔCt method.

### 5.5. Statistical Analysis

Results are expressed as mean ± SD, statistical significance was calculated using Student *t* test for independent samples (SPSS v.15 for Windows), the Levene's Test was used to measure the Equality of Variances, and differences were considered statistically significant at *P* < .05. 

The rate of body weight increase was computed using a linear mixed regression model [5]. The slope obtained for each individual mouse was fitted in a linear regression model and then fitted in a mixed regression model of two linear slopes from 30 to 250 days and 250 to 550 days. The model is Body weight = *β*0 + *β*1Diet + *β*2Time + *β*3Young versus Old+ *β*4Diet*Time + *β*5Time*Age (*β*0 = intercept of SRD group from 30 to 250 days; *β*1 = intercept difference of two groups; *β*2 = slope of SRD group from 30 to 250 days; *β*3 = intercept difference of SRD group from 250 to 550 day and 30 to 250 days; *β*4 = slope difference between 2 groups; *β*5 = slope difference from 250 to 550 day and 30 to 250 day).

## Figures and Tables

**Table 1:** Fatty acid composition of standard and low n-3 fatty acid diet expressed as energy percentage.

	**Standard n-3 diet**	**Low n-3 diet**
	**7912 (3.1 Kcal/g)**	**TD 00522 (3.3 Kcal/g)**
	**Energy percentage**	
**Protein^$^**	25	23
**Fats^$^**	17	16.8
**Carbohydrates^$^**	58	60

** 10:0 **	0.00	0.00
** 12:0**	0.00	0.01
** 14:0**	0.00	0.05
** 16:0**	2.42	1.84
** 18:0**	0.10	0.07
** 20:0**	0.07	0.07
** 22:0**	0.06	0.05
** 24:0**	0.04	0.03
** 16:1**	0.02	0.02
**18:1n9**	3.91	2.78
**18:1n7**	0.16	0.10
**20:1n9**	0.07	0.06
**22:1n9**	0.02	0.01
**24:1n9**	0.00	0.02
**18:2n6**	9.68	12.31
18:3n6*	0.00	0.00
**20:2n6**	0.01	0.01
20:3n6*	0.00	0.00
20:4n6*	0.00	0.00
22:2n6*	0.00	0.00
22:4n6*	0.00	0.00
**18:3n3**	1.02	0.16
20:5n3*	0.00	0.00
22:6n3*	0.00	0.00

*Fatty acid concentration is below 0.01 energy %.

^$^Values were provided by Supplier. Values for individual fatty acids represent the average of three measurements and are expressed as energy %.

**Table 2:** The effect of low n-3 diet on organ and body weight of five months old male mice. The data are expressed as mean ± SD and statistical significance was estimated by using *t*-test, *P* < .03.

	Standard n-3 *n* = 6	Low n-3 *n* = 10
Heart (mg)	197 ± 28.3	230 ± 25.2*
Kidney (mg)	433 ± 132	570 ± 81.2*
Spleen (mg)	141 ± 31.1	147 ± 22.4
Body weight (g)	34.3 ± 3.4	47.1 ± 4.5*

*P < .03.

**Table 3:** The effect of long-lasting low n-3 fatty acid diet on mouse liver fatty acid concentrations in total lipid and total phospholipid extracts. The results are expressed as mean ± SD of 5 measurements per group.

Fatty acids in total lipid and total phospholipid liver extracts (*μ*g/mg tissue)				
	Total Lipids		Total Phospholipids	
	Standard n-3	Low n-3 FA	Standard n-3	Low n-3 FA
**sum saturates**	**15.2 ± 3.2**	** 18.7 ± 8.1**	**2.72 ± 0.7**	**1.98 ± 0.6**
**sum monosats.**	**12.3 ± 7.3**	**21.6 ± 11.6**	**1.24 ± 0.22**	**0.93 ± 0.31**
**18:2n-6**	5.55 ± 1.14	0.23 ± 0.22	1.34 ± 0.49	0.97 ± 0.36
**18:3n-6**	0.088 ± 0.02	0.39 ± 0.34	0.020 ± 0.008	0.017 ± 0.007
**20:2n-6**	0.17 ± 0.046	0.66 ± 0.61	0.041± 0.018	0.030 ± 0.015
**20:3n-6**	0.70 ± 0.11	1.16 ± 0.70	0.17 ± 0.059	0.13 ± 0.063
**20:4n-6**	5.54 ± 0.38	6.64 ± 1.0	1.64 ± 0.55	1.36 ± 0.61
**22:2n-6**	0.13 ± 0.008	0.077 ± 0.065	0.00 ± 0.00^*π*^	0.00 ± 0.00^*π*^
**22:4n-6**	0.18 ± 0.05	0.68 ± 0.48	0.033 ± 0.016	0.037 ± 0.021
**18:3n-3**	0.087 ± 0.037	0.068 ± 0.061	0.01 ± 0.004	0.00 ± 0.00*
**20:3n-3**	0.012 ± 0.007	0.011 ± 0.013	0.002 ± 0.001	0.001 ± 0.001
**20:5n-3**	0.097 ± 0.013	0.027 ± 0.015*	0.022 ± 0.0014	0.00 ± 0.00*
**22:5n-3**	0.17 ± 0.04	0.055 ± 0.025*	0.037 ± 0.017	0.01 ± 0.004*
**22:6n-3**	2.37 ± 0.27	0.78 ± 0.093*	0.73 ± 0.34	0.24 ± 0.08*
**sum n-6^*ν*^**	**6.39 ± 0.47**	**9.22 ± 2.75**	**1.89 ± 0.63**	**1.55 ± 0.68**
**sum n-3^*ν*^**	**2.65 ± 0.26**	0.88 ± 0.14*	**0.80 ± 0.37**	0.25 ± 0.085*
**% n-6 HUFA**	**70.7 ± 2.55**	91.1 ± 1.28*	**70.3 ± 8.6**	84.7 ± 6.5*

^*ν*^Sum n-6 and sum n-3 include the critically bioactive 20 and 22 carbon fatty acids only. The n-6 % HUFA is calculated as: (sum n-6)/[(sum n-6)/(sum n-3)]. 100.

^*π*^Concentrations are lower than 0.01. The statistical significance was calculated using an independent samples T-test as described in methods (Sig. 2-tailed: *P* < 0.05).

*P < 0.05.

**Table 4:** The effect of low n-3 fatty acid diet on stearyl-coenzyme A desaturase 1 (Scd1) and stearyl-coenzyme A desaturase 2 (Scd2) gene regulation in mouse liver. Rt-PCR was performed on total RNA extracts from mouse livers (SABiosciences, Frederick, MD). Ct values corrected against house keeping gene, beta actin, are expressed as average of 4 measurements per group.

Gene	∆Ct Standard n-3	∆Ct Low n-3	Fold Change
Scd1	−2.55	−5.24	6.46
Scd2	10.98	10.83	1.1
